# Stigma perception and health fatalism in parents of children with epilepsy: A cross-sectional study

**DOI:** 10.1016/j.heliyon.2024.e35525

**Published:** 2024-08-03

**Authors:** Mehmet Bulduk, Veysel Can

**Affiliations:** Van Yuzuncu Yil University, Faculty of Health Sciences, Department of Pediatric Nursing, 65000, Van, Turkey

**Keywords:** Epilepsy, Stigma, Health fatalism

## Abstract

**Aim:**

The aim of this study was to examine the factors that affect stigma perceptions and health fatalism of parents of children with epilepsy in eastern Turkey, the relationship between these and the impact of these on their social lives.

**Method:**

This descriptive and cross-sectional study was conducted between August 2022 and January 2023 with the parents of children under the age of 18 who had been diagnosed with epilepsy for at least 1 year and who were followed up in the only hospital with a paediatric neurology outpatient clinic in Van province of Turkey. No sample selection was made in the study. Healthy parents (n = 123) who presented to the outpatient clinic within the specified time period and who agreed to participate in the study after being explained the purpose of the study participated in the study.

**Results:**

In this study, parental age was found to have a statistically weak positive correlation with Health Fatalism Scale (HFS) (r = 0.251; p = 0.005). A weak positive correlation was also found between the years patients had epilepsy and Parent Stigma Scale (PSS) (r = 0.275; p = 0.002). In addition, a statistically positive and weak relationship was found between Parent Stigma Scale scores and Health Fatalism Scale scores (r = 0.212; p = 0.018). This study found significant relationships between stigma perception and health fatalism in parents of epileptic children. Stigma perception increased with disease duration and lower parental education levels.

**Conclusion:**

While providing an important basis for understanding the difficulties experienced by parents and developing support mechanisms, the present study can contribute to more informed support for parents of patients with epilepsy in the community. Nurses can contribute to ending stigma and discrimination by identifying patients' and parents’ perceptions of epilepsy, focusing on addressing gaps in knowledge and raising awareness in the community.

## Highlights

• Parents with low education and income perceive stigma and health fatalism.
• Most parents see discrimination against their epileptic kids, harming health.
• Stigma and stress increase with epilepsy duration, especially in severe cases.

## 1 Introduction

Epilepsy is a common neurological disorder affecting approximately 50 million people worldwide, with an onset mostly in childhood and often associated with cognitive and behavioural complications [[1], [2], [3]] (see Table 1, Table 2A, Table 2B, Table 3, Table 4, Table 5).

**Table 1:** Frequency distributions of the demographic variables.

	Frequency	Percentage
Sex of the parent		
Female	85	69.1
Male	38	30.9
Marital Status		
Single	0	0
Married	123	100
Educational Status		
Elementary	64	52
Secondary	30	24.4
High school	23	18.7
University	6	4.9
Monthly income		
Income < expense	84	68.3
Income = expense	38	30.9
Income > expense	1	0.8
Type of family		
Nuclear	84	68.3
Extended	39	31.7
Place of residence		
Urban	70	56.9
Rural	53	43.1
Employment status		
Employed	32	26
Unemployed	91	74
Sex of the child		
Female	58	47.2
Male	65	52.8
Total years the child has been epilepsy patient		
1–3 Years	49	39.8
3–5 Years	38	30.9
>5 Years	36	29.3
Total years the child has had seizures		
<1 Year	5	4.1
1–3 Years	81	65.9
3–5 Years	30	24.4
>5 Years	7	5.7
Seizure frequency of the child		
No seizures	72	58.5
Once a month	36	29.3
A few times a month	15	12.2

Parent's occupation		
Housewife	79	64.2
Tradesman	30	24.4
Civil servant	5	4.1
Unemployed	4	3.3
Health professional	2	1.6
Educator	2	1.6
Religious official	1	0.8
What are your thoughts on the social activities/relationships of a child with epilepsy^a^		
Can attend a normal school	94	79.7
Can be ashamed due to the disease	59	50
Has difficulty in making friends	20	16.9
Should be isolated	19	16.1
Has lower life expectancy	16	13.6
Has difficulty in being successful in life	15	12.7
Cannot have normal social roles	9	7.6
Cannot get married	2	1.7
Cannot have children	2	1.7
Can be identified at first sight	1	0.8
What are your thoughts on the cause of epilepsy^a^		
It is a neurological disease	106	86.2
It is a serious psychiatric disease	29	23.6
It is a brain damage	23	18.7
It is a mental disorder	18	14.6
It is genetic	18	14.6
It results from depression	18	14.6
It is a supernatural disease	12	9.8
Consanguineous marriage	9	7.3
It is a sign of physical deficiency/weakness	7	5.7
It is a punishment for past sins/past life.	1	0.8
Faith can heal epilepsy		
No	31	25.2
Yes	92	74.8
Do you tell people about your child's disease		
Undecided	10	8.1
I usually tell	63	51.2
I usually do not tell	50	40.7
	Mean.± sd	Mean (min - max)
Parent's age	38.19 ± 8.18	38 (19–63)
Total years the child has been epilepsy patient	4.21 ± 3.09	4 (1–17)

a Multiple answers.

**Table 2A:** Analysis of the relationship between quantitative data and scale scores.

		PSS	HFS
Parent's age	r	−0.048∗	0.251∗
	p	0.598	**0.005**
How many years the patient has had epilepsy	r	0.275∗∗	0.033∗∗
	p	**0.002**	0.716

∗ Pearson Correlation Coefficient.

∗∗ Spearman's rho Correlation Coefficient.

**Table 2B:** Analysis of the correlation between scale scores.

		HFS
PSS	r	0.212
	p	**0.018**

r: Pearson Correlation Coefficient.

A statistically positive and weak relationship was found between Parent Stigma Scale score and Health Fatalism Scale scores. (r = 0.212; p = 0.018).

**Table 3:** Comparison of scale scores in terms of demographic data.

	PSS		Test Sta.	p	HFS		Test Statistics	p
	Mean ± sd	Mean (min-max)			Mean ± sd	Mean (min-max)		
Parent's sex								
Female	3.26 ± 1.2	3.6 (1–5)	t = 2.573	**0.011**	61.42 ± 15.52	59 (17–85)	t = 0.652	0.516
Male	2.65 ± 1.28	2.8 (1–5)			59.42 ± 16.22	57.5 (21–85)		
Educational status								
Elementary	3.23 ± 1.19^b^	3.6 (1–5)	F = 4.476	**0.009**	63.11 ± 13.89^b^	61.5 (21–85)	F = 2.828	**0.042**
Secondary	3.18 ± 1.3^ab^	3.4 (1–5)			61.87 ± 17.49^b^	58 (17–85)		
High school	2.64 ± 1.43^ab^	2.8 (1–5)			56.78 ± 17.19^ab^	54 (21–85)		
University	2.5 ± 0.37^a^	2.5 (2–3)			46.33 ± 10.63^a^	47.5 (34–62)		
Type of family								
Nuclear	3.16 ± 1.26	3.5 (1–5)	t = 1.184	0.239	61.83 ± 16.09	61 (17–85)	t = 1.067	0.288
Extended	2.88 ± 1.25	2.8 (1–5)			58.59 ± 14.79	58 (21–85)		
Place of residence								
Urban	2.82 ± 1.22	2.8 (1–5)	t = −2.633	**0.010**	58.34 ± 15.86	56 (21–85)	t = −2.023	**0.045**
Rural	3.41 ± 1.23	3.8 (1–5)			64.06 ± 15.04	63 (17–85)		
Employment status								
Employed	2.73 ± 1.21	2.7 (1–5)	t = −1.842	0.068	55.47 ± 15.2	54 (21–85)	t = −2.272	**0.025**
Unemployed	3.2 ± 1.25	3.4 (1–5)			62.68 ± 15.52	61 (17–85)		
Sex of the child								
Female	3.29 ± 1.12	3.4 (1–5)	t = 1.873	0.064	60.4 ± 14.68	57.5 (17–85)	t = −0.271	0.787
Male	2.88 ± 1.34	3.2 (1–5)			61.17 ± 16.67	61 (21–85)		
Total years the child has been epilepsy patient								
1–3 Years	2.73 ± 1.18^a^	2.6 (1–5)	F = 3.732	**0.027**	60.63 ± 14.82	59 (21–85)	F = 0.069	0.934
3–5 Years	3.15 ± 1.35^ab^	3.5 (1–5)			61.61 ± 19.12	63 (17–85)		
>5 Years	3.46 ± 1.15^b^	3.7 (1–5)			60.19 ± 13.07	57 (35–85)		
Seizure frequency of the child								
No seizures	3.13 ± 1.37	3.4 (1–5)	F = 0.647	0.529	61.51 ± 17.64	57.5 (17–85)	F = 0.141	0.869
Once a month	2.9 ± 1.15	2.8 (1–5)			59.92 ± 13.4	61.5 (21–85)		
A few times a month	3.23 ± 0.93	3.6 (1.4–4.2)			60.43 ± 10.62	58.5 (41–84)		
Faith can heal epilepsy								
No	2.75 ± 1.41	3.2 (1–5)	t = −1.678	0.096	58.06 ± 17.9	53 (21–85)	t = −1.125	0.263
Yes	3.18 ± 1.19	3.4 (1–5)			61.73 ± 14.89	61 (17–85)		
Do you tell people about your child's disease								
Undecided	2.62 ± 1.32^a^	2.5 (1–5)	F = 10.018	<**0.001**	62.7 ± 13.12	57.5 (47–85)	F = 2.97	0.055
I usually tell	2.69 ± 1.14^a^	2.6 (1–5)			57.52 ± 14.52	57 (21–85)		
I usually do not tell	3.64 ± 1.19^b^	4.2 (1–5)			64.56 ± 16.93	63 (17–85)		

t: Independent Samples *t*-test: F: One-way Analysis of Variance; Mean ± Standard Deviation; Median (minimum - maximum); a-b: There is no difference between groups with the same letter.

**Table 4:** Analysis of independent variables affecting PSS by Robust Regression Analysis.

	β0 (95 % CI)	S. Error	β1	t	p	VIF
Fixed	2.039 (0.944–3.134)	0.553		3.689	<**0.001**	
HFS	0.01 (−0.003–0.023)	0.006	0.124	1.504	0.135	1.142
Educational status (Elementary)	Reference					
Secondary	0.052 (−0.405–0.509)	0.230	0.019	0.227	0.821	1.130
High school	−0.284 (−0.869–0.3)	0.295	−0.091	−0.964	0.337	1.503
University	0.23 (−0.814–1.274)	0.527	0.042	0.436	0.663	1.555
Monthly income (Income < expense)	Reference					
Income = expense	−0.87 (−1.302–−0.438)	0.218	−0.335	−3.994	<**0.001**	1.182
Income > expense	−0.999 (−3.225–1.228)	1.123	−0.076	−0.889	0.376	1.232
Type of family (Nuclear)	Reference					
Extended	−0.122 (−0.54–0.295)	0.211	−0.047	−0.581	0.562	1.105
Place of residence (Urban)	Reference					
Rural	0.507 (0.109–0.905)	0.201	0.208	2.522	**0.013**	1.142
Employment status (Employed)	Reference					
Unemployed	0.046 (−0.498–0.591)	0.275	0.017	0.168	0.867	1.690
Total years the child has been epilepsy patient (1–3)	Reference					
3–5	0.533 (0.069–0.996)	0.234	0.202	2.277	**0.025**	1.327
>5	0.823 (0.359–1.287)	0.234	0.312	3.513	**0.001**	1.326
Faith can heal epilepsy (No)	Reference					
Yes	0.254 (−0.198–0.705)	0.228	0.091	1.114	0.268	1.117

F = 4.878, p < 0.001, R^2^ = 34.7 %, β0: Unstandardized beta coefficient, β1: Standardized beta coefficient.

**Table 5:** Analysis of independent variables affecting HFS by Robust Regression Analysis.

	β0 (95 % CI)	S. Error	β1	t	p	VIF
Fixed	58.562 (47.914–69.211)	5.374		10.898	<**0.001**	
Educational status (Elementary)	Reference					
Secondary	0.818 (−5.428–7.064)	3.152	0.024	0.259	0.796	1.147
High school	−2.714 (−10.59–5.162)	3.975	−0.072	−0.683	0.496	1.489
University	−11.526 (−25.444–2.392)	7.024	−0.175	−1.641	0.104	1.524
Monthly income (Income < expense)	Reference					
Income = expense	−3.752 (−9.524–2.02)	2.913	−0.119	−1.288	0.200	1.153
Income > expense	−0.128 (−30.109–29.852)	15.130	−0.001	−0.008	0.993	1.232
Type of family (Nuclear)	Reference					
Extended	−4.617 (−10.205–0.972)	2.820	−0.148	−1.637	0.104	1.103
Place of residence (Urban)	Reference					
Rural	5.188 (−0.127–10.503)	2.682	0.177	1.934	0.056	1.122
Employment status (Employed)	Reference					
Unemployed	3.289 (−4.071–10.649)	3.714	0.099	0.886	0.378	1.691
Total years the child has been epilepsy patient (1–3)	Reference					
3–5	2.014 (−4.264–8.293)	3.168	0.064	0.636	0.526	1.343
>5	−1.889 (−8.147–4.369)	3.158	−0.059	−0.598	0.551	1.327
Faith can heal epilepsy (No)	Reference					
Yes	2.092 (−3.898–8.082)	3.023	0.062	0.692	0.490	1.082

F = 2.133, p = 0.023, R^2^ = 17.4 %, β0: Unstandardized beta coefficient, β1: Standardized beta coefficient.

Epilepsy is a disease that significantly affects quality of life through clinical variables such as age of onset, duration, type, severity and frequency of seizures [4]. While childhood epilepsy has adverse effects on physical and psychological health as well as social and professional domains, as reported in the literature, this condition is also associated with a marked increase in the risk of somatic, nutritional, infectious, neurological, psychiatric, cognitive status, behavioural problems, anxiety and depression [[5], [6], [7], [8]].

Fatalism is a common way of thinking in Turkey [9]. Studies evaluating attitudes towards epilepsy and health fatalism have been conducted in various regions of Turkey [[10], [11], [12], [13]]. In a meta-analysis of 46 studies on the relationship between fatalism and health behaviours, a positive correlation was found between high levels of fatalism and health-threatening behaviours [14]. It is stated that health fatalism negatively affects health behaviours of individuals and prevents their active participation in the diagnosis and treatment process [15]. Individuals with high fatalism tendency display reluctant behaviours in planning for the future, make limited efforts to achieve desired goals and generally adopt a passive attitude towards external factors [16].

Problems of parents and children often interact [17]. Paediatric patients should be evaluated together with their parents in coping with chronic diseases [18,19]. Parents of a child with epilepsy are at significant risk of psychological problems such as depression, anxiety and increased stress [20].

Stigma is a global phenomenon with significant negative impacts on health-related quality of life in patients with epilepsy and their families [21,22]. Parents’ perceptions of stigmatisation of their children with epilepsy are also significantly associated with psychopathology in children with epilepsy [23,24]. Social stigmatisation, which leads to discrimination of individuals with epilepsy in employment, education and social environment, has serious psychological and/or emotional consequences for families and caregivers [4]. It is important to evaluate knowledge about epilepsy and attitudes towards epilepsy in order to fight social stigmatisation and discrimination [10]. When a child gets sick, many factors such as the values and sociocultural structure of the parents play an active role in the diagnosis and treatment process [25].

It is important to study the effects of fatalism, which is widely accepted in Turkey, on attitudes towards epilepsy and stigmatisation. In addition to negatively affecting individuals' health behaviours and coping skills, fatalism may also make the stigmatisation perception of children's parents more intense and severe. The main hypothesis of our study is that parents with high fatalistic dispositions tend to perceive epilepsy-related stigmatisation and discrimination more intensely. By examining this relationship, we aim to contribute to the development of awareness and support programs for children with epilepsy and their families.

## 2 Method

### 2.1 Type of research

The study was conducted with a descriptive and cross-sectional design.

### 2.2 Place and time of research

This study was conducted between August 2022 and January 2023 with the parents of children under 18 years of age who were diagnosed with epilepsy for at least 1 year and who were followed up in the paediatric neurology outpatient clinic of a secondary care hospital in Van province of Turkey. No sample was selected in the study. All parents who presented to the outpatient clinic within the specified time period and who agreed to participate in the study (n = 123) were included in the study.

### 2.3 Inclusion criteria

Parents.

• who had a child diagnosed with epilepsy between the ages of 0–18 years,
• who completed and signed the informed consent form before participating in the study,
• who could speak Turkish so that they could understand the survey and evaluation tools,
• who were 18 years of age or older

were included in the study.

### 2.4 Exclusion criteria

Parents.

• whose children had severe and chronic diseases other than epilepsy (e.g. cancer, serious heart diseases)
• who had an active, untreated serious psychiatric diagnosis such as schizophrenia or bipolar disorder
• who had cognitive impairment or dementia to prevent from understanding and participating effectively in the survey completion and evaluation processes
• who were in the last stage of their pregnancy and at risk of experiencing additional stress and health problems during this period
• who were participating in another similar study at the same time period

were excluded from the study.

## 3 Data collection tools

The study data were collected through face-to-face interview by using an 18-question “Survey Form” prepared by the researcher in line with the literature [[26], [27], [28], [29]]. The survey form consisted of questions to determine the sociodemographic characteristics of the patients and parents in the study group, how many years the patients have had epilepsy, how long they have had seizures and the frequency of seizures, the cause of epilepsy and the relationship between faith and epilepsy.

**Parent Stigma Scale**; Parent Stigma Scale was developed by Austin et al., in 2004 to determine how parents of epileptic patients compare their children with others. This scale consists of five items that can be answered in five-point Likert type (1-Strongly disagree, 2-Disagree, 3- Cannot decide, 4-Agree and 5- Strongly agree). The mean score is obtained by dividing the total score of each item by the total number of items (n = 5). High mean scores show high stigma perception and according to Austin et al., the Cronbach α coefficient of the scale is 0.79 [27]. The validity and reliability of the Turkish version of Parent Stigma Scale was conducted by Köse et al. Cronbach's α coefficient was found to be 0.87 [28]. In this study, Cronbach's α coefficient was found as .0.82.

**Health Fatalism Scale;** Health Fatalism Scale developed by Franklin, Schlundt and Wallson [30] was used as data collection tool. This scale consists of five items that can be answered in five-point Likert type (1-Strongly disagree, 2-Disagree, 3-Undecided, 4-Agree and 5-Strongly agree). Turkish version of the scale consists of one dimension and 17 items. The minimum possible score of the scale is 17 and the maximum possible score is 85. As the scale score increases, fatalism also increases. Bobov and Çapık reported the Cronbach's α coefficient as 0.91 in the Turkish validity and reliability study [26]. In this study, Cronbach's α coefficient was found as .0.91.

**Data Collection;** Before the data were collected, the purpose of the study was explained verbally and in writing to all parents who agreed to participate in the study and their consent was obtained. Data were collected in the Child Neurology outpatient clinic through individual face-to-face interviews of 15–20 min.

### 3.1 Evaluation of data

The data were analysed with IBM SPSS V23. Skewness and Kurtosis coefficients were used to analyse conformity to normal distribution. Independent Samples *t*-Test was used to compare the scale scores conforming to normal distribution in paired groups. One-way Analysis of Variance was used to compare the scale scores conforming to normal distribution in groups of three or more, and multiple comparisons were made with Duncan and Tamhane Tests. Pearson's correlation coefficient was used to analyse the relationship between the normally distributed variables and the scale scores, and Spearman's rho correlation coefficient was used to analyse the relationship between non-normally distributed variables and the scale scores. The results of the analyses were presented as mean ± standard deviation and median (minimum - maximum). Significance level was taken as p < 0.050. In this study, normality test coefficient was based on ±2 [31]. Data were analysed with R software for regression analysis. Compliance to normal distribution was analysed by Kolmogorov-Smirnov Test. Robust Regression Analysis was used to examine the independent variables affecting the Parent Stigma Scale score and Health Fatalism Scale score, which did not fit the normal distribution. Significance level was taken as p < 0.050.

### 3.2 Strengths and limitations of the research

The fact that the data collection tool is based on self-report can be considered as a limitation of this study. The study was conducted in only one province in eastern Turkey. Therefore, it is not possible to generalise the results to the whole of Turkey.

### 3.3 Ethical considerations

Prior to the research, approval was obtained from the Van Yüzüncü Yıl University Non-Interventional Clinical Research Ethics Committee on 20/05/2022 (2022/5–09 approval number). In addition, official written informed consent was received from the hospital where the study was conducted. During the collection of research data, the principles of the Declaration of Helsinki, scientific ethics and data confidentiality principles were adhered to.

## 4 Results

Majority of the parents in the study are women and all participants are married. In terms of education level, most of the participants are elementary school graduates. Most of the participants reported low income and the majority of them have a nuclear family structure. More than half of the participants live in urban areas. The rate of working parents is low and the majority of the participants are housewives. Sex distribution of children is balanced.

In terms of duration of epilepsy, a significant number of participants stated their children have had epilepsy between 1 and 3 years. Most of the parents stated that their children did not have seizures. The mean age of the participants is 38 years and the mean duration of epilepsy is 4 years. The majority of parents think that children with epilepsy can attend regular school. Most of the participants think that epilepsy is a neurological disease and a significant number of them believe that it can be cured with faith. There is also a significant number of respondents who told others about their child's condition.

A statistically weak positive correlation was found between parent's age and HFS (r = 0.251; p = 0.005). A statistically weak positive correlation was found between the duration of epilepsy and the Parent Stigma Scale (r = 0.275; p = 0.002).

According to the results of the study, significant differences were found between parents' sex (p = 0.011) and educational level (p = 0.009) and stigma scale scores, and between educational level (p = 0.042), place of residence (p = 0.045) and employment status (p = 0.025) and health fatalism scale scores. In addition, a significant difference was found between the place of residence and the stigma scale scores (p = 0.010). The status of telling the child's illness to other people affected the stigma scores (p < 0.001). No significant difference was found in other demographic characteristics (p > 0.050).

The independent variables affecting the PSS score were analysed by Robust Regression Analysis and the model was found to be statistically significant (F = 4.878; p < 0.001). The Parent Stigma Scale score of the participants whose income was equal to their expenses was 0.870 units lower than those whose income was lower than their expense (p < 0.001). PSS score of participants living in rural areas was 0.507 units higher than those living in urban areas (p = 0.013). PSS score of participants whose children had epilepsy for 3–5 years was 0.533 units higher than those whose children had epilepsy for 1–3 years (p = 0.025). PSS score of participants whose children had epilepsy for 5 years or more was 0.823 units higher than those whose children had epilepsy for 1–3 years (p = 0.001). There was no statistically significant effect of the participants’ HFS score and other variables on the PSS score (p > 0.050).

The independent variables affecting the HFS score were analysed by Robust Regression Analysis and the model was found to be statistically significant (F = 2.133; p = 0.023). When the independent variables included in the model were analysed, no significant variable was found to affect the HFS score (p > 0.050).

## 5 Discussion

This study was conducted to determine the relationship between stigma perceptions and health fatalism of parents of children with epilepsy. Research shows that parents of children with epilepsy perceive different levels of stigma and that this stigma perception harms children and their families as much as the disease itself [32,33] However, the reasons for this stigmatisation have not been fully clarified [34]. Research on the stigma perception and health fatalism of parents of children with epilepsy is important. However, a comprehensive study on this subject has not been conducted yet. More than half of the parents think that individuals with epilepsy are discriminated against [35]. Negative effects of chronic diseases on parents’ mental health are well known [36]. It is also reported that individuals with more negative attitudes towards epilepsy have higher levels of health fatalism [13]. Stigma perception of parents towards their children with epilepsy is also significantly associated with psychopathology in children with epilepsy [23,24]. In our study, a weak statistically positive relationship was found between PSS and HFS scores of children with epilepsy. However, the weak level of the relationship and the lack of a link between these factors as a result of further analyses show that the relationship between these is not direct and significant. These findings emphasise the complexity of the experiences of parents and require more in-depth research.

Children living with epilepsy are at high risk for behavioural problems such as learning disorders, inattention, hyperactivity and psychiatric disorders such as anxiety, depression and psychosis [37]. Therefore, epilepsy affects not only children but also their parents significantly. Most parents are not aware of the real risks associated with epilepsy. Despite having adequate knowledge, attitudes and practice regarding epilepsy in children, many parents fail to take appropriate measures for various reasons [35]. In this context, it is possible that children with epilepsy may face lifelong difficulties unless a deliberate intervention is made to reduce the impact of factors considered to be challenging and to meet their needs [38]. In our study, it was found that having epilepsy for a long time had a negative effect on the parents and there was a statistically positive and weak relationship between the duration of the patient's epilepsy and the Parent Stigma Scale.

In our study, the result that parents of children who had epilepsy for more than five years had higher parent stigma scale scores compared to other groups, which was statistically significant. This finding suggests that prolonged duration of epilepsy may increase stigma perception of parents. Parental stress may be more severe in untreatable epilepsy [39]. In fact, although most children with epilepsy have good seizure control, about 20 % continue to have seizures despite treatment [39,40]. Furthermore, it has also been shown that the severity of epilepsy, particularly in children with drug-resistant epilepsy, significantly affects parenting stress over time, which is indicative of an increasing burden as the severity of the condition persists. This suggests a lasting effect of stigma over time [41]. It further suggests that stigma has a lasting impact on the well-being of parents over time, emphasising that parental stress has important implications for overall clinical care and that understanding the relevant sources of this stress can improve outcomes for children with epilepsy [42]. As a result, stigma perception among parents of children with epilepsy tends to increase over time, influenced by factors such as the severity of epilepsy, lack of public knowledge and constant stress of parents. In our study, a statistically weak positive correlation was found between parental age and HFS. It is stated that as the age increases, the attitude towards epilepsy becomes more negative and health fatalism gets higher [13]. This can also be explained by the fact that the ageing of humans and the tendency towards spirituality are related [12]. In the process of ageing, there may be a decrease in physical strength and changes in the ability to cope with the difficulties of life. This may cause individuals to have increased concerns about the future of their children.

Stigma experienced by parents of children with epilepsy is a complex and multifaceted issue. Although direct research comparing the stigma experienced by mothers and fathers of children with epilepsy is limited, available evidence suggests that both groups of parents experience high levels of stress, anxiety and psychopathological reactions [39,43,44]. It has been reported that parents of children with epilepsy often experience high levels of stress and perceived stigma [45]. A statistically significant difference was found between the mean parent stigma scale scores in terms of the sex of parents. Stigma depends on various factors such as social isolation, marital problems and anxiety [46]. Fear of stigma is the main reason for not reporting this situation to friends [47].

This study shows that mothers have higher mean stigma scale scores than fathers. This may be related to the fact that 64 % of the parents are housewives, which leads to social isolation. This isolation may have contributed to the increase in stigma experienced by mothers of children with epilepsy by preventing them from receiving social or professional support. Studies show that participants with higher levels of education and income have more knowledge about epilepsy [[48], [49], [50]]. While this situation leads to a decrease in negative attitudes towards the disease and an increase in positive attitudes, it also shows that stigmatisation levels towards epilepsy have decreased. A statistically significant difference was found between the mean values of the parent stigma scale score in terms of education level. Here, a difference was found between the scores of primary school graduates and university graduates. The mean stigma scale score was higher in elementary school graduates. Our research supports the view that the stigma levels of parents of children with epilepsy decrease as the level of education increases [14,51]. This information emphasises the importance of considering parents’ educational status in addressing stigma and promoting better outcomes for both parents and children affected by epilepsy. In our study, HFS score in terms of educational status was higher in individuals with low education level. There are evaluations which show that there is a strong relationship between the educational level of parents and their health beliefs, attitudes and behaviours. Individuals with lower levels of education are generally observed to have lower health literacy, higher levels of health fatalism, and more prevalence of dysfunctional attitudes and cognitive-behavioural avoidance tendencies. This emphasises the impact of education level on health-related beliefs and behaviours [[52], [53], [54], [55], [56], [57]]. There is a strong relationship between parental education and health fatalism, especially among parents with lower levels of education. This increases the likelihood that children will show a higher predisposition to levels of health fatalism that may affect health outcomes.

Children of highly educated parents have more physical activity and shorter screen time, suggesting that parents may be more aware of health issues [58]. Individuals with low levels of education may experience difficulties in accessing the necessary resources to achieve their health goals. This may trigger feelings of powerlessness, fatalism and helplessness [59]. Evidence from relevant sources supports the claim that parents with lower levels of education are more likely to have health fatalism, which can have important implications for the health of their children [60]. Low stigma and health fatalism levels observed in urban areas are usually thought to be associated with factors such as high levels of education, effective social support mechanisms and wide access to information.

In our study, the monthly income of most of the participants was lower than their expenses. A statistically significant difference was found in the mean HFS scores of those who were not working in terms of employment status. Previous studies conducted in Turkey have shown that individuals with lower income have higher mean scores of health fatalism [26,61]. Similarly, individuals living in extreme poverty or destitution in South America have higher fatalism scores [62]. Likewise, in the developed countries of the European Union, people often have a fatalistic perception regarding the causes of poverty [63]. Research has shown that individuals with low income also have higher levels of fatalism towards their health.

It has been shown that parents of children with epilepsy may perceive negative reactions from others and limit family social interaction, which can lead to emotional reactions such as anger, guilt, fear, anxiety and depression [45]. It has also been shown that closed communication about epilepsy is associated with poorer psychosocial well-being in children living with epilepsy and their parents [29]. Kampra et al. found that parents of children with epilepsy experienced high levels of difficulty and that parents concealed their children's illnesses due to anxiety, shame and fear of stigma. In the study, it was also emphasised that these attitudes shown consciously or unconsciously by parents are role models for children in the perception of the disease [64]. These results suggest that parents of children with epilepsy may experience stress, psychopathology and difficulties in explaining their child's condition, which may affect their decision to conceal the disease from others. As a matter of fact, in our study, the stigma scale scores of parents who usually did not tell others about their child's disease were found to be higher.

Having information about epilepsy can increase the coping abilities of patients and their families and positively affect their social-psychological functioning [65]. Therefore, healthcare professionals, especially nurses, have an important role in reducing stigma associated with epilepsy. This responsibility includes organising actions against stigmatisation and discrimination to ensure that negative perceptions of individuals with epilepsy are changed. The first step is to identify patients' and parents’ perceptions of epilepsy [28]. Such research provides an important basis for understanding the challenges faced by parents and for developing support mechanisms. In this way, it may be possible to support the community in a more informed way and provide more effective assistance to parents.

## 6 Conclusion

This study examined the relationship between the stigma perception and health fatalism of parents of children with epilepsy. The relationship between health fatalism and stigma was found to be weak. However, this relationship was not direct and significant. The study also showed that education level, income level and family type affected these perceptions. It was found that most of the parents experienced perceptions of discrimination and had negative feelings about their children with epilepsy. It is important for nurses to develop support mechanisms and provide guidance to families. By identifying patients' and parents’ perceptions of epilepsy, they can contribute to ending stigmatisation and discrimination through their efforts to change these perceptions.

## Data availability statement

Data will be made available on request.

## Funding

This research did not receive any specific grant from funding agencies in the public, commercial, or not-for-profit sectors.

## CRediT authorship contribution statement

**Mehmet Bulduk:** Writing – review & editing, Writing – original draft, Visualization, Validation, Supervision, Software, Resources, Project administration, Methodology, Investigation, Funding acquisition, Formal analysis, Data curation, Conceptualization. **Veysel Can:** Writing – review & editing, Data curation.

## Declaration of competing interest

The authors declare that they have no known competing financial interests or personal relationships that could have appeared to influence the work reported in this paper.
